# Reports of Cases Occurring in the Medical Ward of the Buffalo Hospital

**Published:** 1857-06

**Authors:** A. W. Nichols

**Affiliations:** Assistant to Chhair of Clinical Medicine, University of Buffalo


### BUFFALO MEDICAL JOURNAL

AND

### MONTHLY REVIEW.

**VOL. 13.** JUNE, 1857. **NO. 1.**

### ORIGINAL COMMUNICATIONS.

ART. I.—*Reports of Cases treated in the Male Medical Ward of the Buffalo Hospital of the Sisters of Charity*, AUSTIN FLINT, M. D., *Attending Physician, during the College Session of* 1856–7, *being from October* 1, 1856 *to February* 18, 1857. Reported by A. W. NICHOLS, M. D., Assistant to Chhair of Clinical Medicine, University of Buffalo.

### No. III. *Emphysema: Asthma: Plastic or Pseudo-membranous Bronchitis.*

Three cases of emphysema treated in the ward, were made the subject of clinical remarks during the past college session; the first case was not complicated at its commencement to any appreciable degree, with bronchial disease; the second was accompanied by chronic bronchitis, and occurred in a person who had had empyema forty-three years pri r to admission into hospital; the third was attended by well-marked paroxysms of asthma and by a rare variety of bronchitis, namely, plastic or pseudo-membranous, in which false membranes in the forms of casts of the bronchior in their shreds, are expelled.

Case I. Was an instance of emphysema, limited to the upper lobes of the right lung and accompanied by very slight bronchial affection.

P. D. Irish, aged 23, laborer; had intermittent fever a year and a half ago, and much of the time since. Never experienced any pulmonary trouble up to six months ago. Was suddenly attacked, whilst at work, with difficulty of breathing. Had *no cough prior* to this attack, nor any up to four months since. The difficulty of breathing gave him scarcely any inconvenience except on active exercise, when he suffered from deficiency of breath. He thinks, the shortness of breath had been progressively increasing since date of attack. He had no want of breath in *early life*, *nor at any time prior* to six months ago. Does not know that either of his parents had any asthmatic affection. The cough has gradually increased; it was slight at first; now, it is violent, occurring oftener at night than during the day; the spasmodic character of the cough could not be determined accurately; although since the case was recorded, the cough has appeared to be paroxysmal. The cough is wholly *unattended* by expectoration. He has had no pain in the chest, nor hæmoptysis, at any time. The spleen is now considerably enlarged, and has been so for six months. His aspect is not morbid; appetite good; bowels habitually constipated. There is considerable capillary congestion of backs of hands and of forearms, darkish in hue: no lividity of lips nor of finger-nails. Pulse 68 and small. Respiration 20.

The physical signs on an examination of the chest, disclosed emphysema affecting the upper lobe of the right lung. They were, briefly, as follows: the superior third of right side of chest in front, evidently fuller than same portion of left side; superior costal type of respiration much more marked on left than on right side. Intercostal depressions, below and laterally, equally marked on the two sides. The resonance everywhere on right side, in front, higher in pitch than on left side and vesiculo-tympanitic in quality; amount of sonorousness about the same on both sides. Respiration, over upper and middle thirds of chest, in front; on left side, inspiratory sound tolerably evolved, vesicular, of normal length, with no expiratory sound; on right side, inspiratory sound fuller, vesicular, but *deferred*, frequently not heard at all, with no expiratory sound. A sibilant rale was heard with expiration, in upper scapular region of right side.

He was placed on the use of potassii iodidum, gr. v., 3 times daily; and in about one month after his admission, was discharged from treatment, reporting much improved. The paroxysms of cough were less frequent and less severe; and there was less deficiency of breath on active exercise. For pathaology and objects of treatment, vide remarks.

CASE II. Was an instance of emphysema, accompanied by chronic bronchitis, and affecting both sides of the chest.

J. W. Swiss, aged 58, weaver; from the history he gives and from the physical signs afforded by an examination of the chest, it appears that when he was fifteen years of age, he had *empyema* affecting the left side of chest; after an illness of ten months, during which time the operation of paracentesis thoracis was performed, he regained his health and continued free from sickness, up to three years ago, working at his loom and on a farm. Three years ago, he had œdema of lower extremities, cough, and pain in the chest. These symptoms disappeared in a short time, but he has had ever since deficiency of breath more troublesome in winter than in summer. He never has had paroxysms of dyspnœa, nor much embarrassment of respiration. He breathes more easily during the night than during the day; preferring a semi-recumbent posture. For three months, he has had cough, and some expectoration: the latter being rather difficult and consisting of transparent, watery mucus, resembling saliva. His aspect was not notably morbid; the cheeks are flushed, the lips of a dull red color; the dorsal surface of hands presents considerable capillary congestion. Respiration 36. Pulse 54.

Physical signs determined the prior existence of empyema, and the presence of emphysema and chronic bronchitis affecting both sides of chest. Inspection determined the type of breathing to be *chiefly abdominal;* but more superior and inferior costal movement on the *right* side than on the left; the intercostal depressions were marked on the anterior and lateral portion of left side; below the level of left nipple, evident flattening and contraction of chest. On percussion, the resonance, on both sides, in front, was clear and vesiculo-tympanitic. On auscultation, on both sides, in front at the upper third of chest, the inspiratory sound was vesicular, rather intense, and *deferred*,* followed by a prolonged and feebler expiratory sound, and a sonorous rale. On the left side, in front and behind, generally, the inspiratory sound was less intense, less vesicular, and more deferred than on the right side. Occasionally, sonorous and sibilant rales on both sides.

The *lac ammoniac* was prescribed and continued until he left the hospital. This medicine gave him marked relief. (The foregoing account was transcribed from the record taken during his sojourn in the ward, from March

---

* Vide " Flint on the Respiratory Organ, p. 446.

to May, 1856.) He reëntered the hospital in November following, complaining of difficult respiration, cough, and expectoration. An examination of the chest, afforded much the same physical signs as have been recounted above.

He was suffering from an aggravation of the chronic bronchitis, with which he had been previously troubled. Sibilant and sonorous rales were present, everywhere on both sides: the impulse of the heart was felt in the epigastrium, alone; and both sounds of the heart were heard there distinctly. He was placed on the use of the potassi iodidum, gr. v., 3 times daily, increased so as to take gr. x., 3 times; and continued it for three or four weeks. It was then discontinued, as he reported not improved; the cough and difficulty of breathing continued, with the lividity of lips and capillary congestion of the backs of the hands. He was then placed on the mistura gum ammoniaci, from which he has obtained much relief. For the past four months, he has been up and about the wards, complaining of cough and deficiency of breath, which at times are so much increased as to compel him to take the bed, and maintain a semi-recumbent posture. From the ammoniac mixture, and a solution of morphia in the syrup of tolu, he has always obtained *much and speedy relief.*

For parthaology and objects of treatment, vide remarks.

Case III. Was an instance of emphysema, accompanied by bronchitis of a rare form, characterized by the expulsion of false membrane from the bronchial tubes, and also attended by well marked paroxysms of asthma.

H. M. German, aged 35, laborer; his father was troubled with paroxysms of asthma; he has been short winded after active exercise, as long as he can remember. He has suffered from attacks of asthma during the past three years only: they have progressively become more and more frequent. He has observed them to occur with changes in the weather. So far as can be ascertained, they are not preceded by cough; and in the intervals, he has not habitual cough. These attacks are followed by cough and by expectoration. At the period of his admission, he was laboring under a severe paroxysm of asthma. He was obliged to maintain the sitting posture in bed; the respiration was labored; the inspiration was shortened and quickened; the expiration labored and prolonged, and accompanied by a peculier wheezing sound; dry rales existed everywhere, on both sides of chest; resonance on percussion, was clear. In the evening following, after expectoration quite copious, he obtained relief, and was much more comfortable the next day. The cheeks were congested and of a dusky hue; the lips were livid; expectoration was difficult. He was obliged to maintain a semi-recumbent posture in bed. The skin was warm and dry. Respiration 20. Pulse 112.

Physical signs afforded the evidence of emphysema affecting the upper lobes of both lungs, more marked on the left than on the right side; and the existence of bronchitis. On inspection, the type of breathing was almost entirely abdominal; the intercostal depressions were marked, laterally and inferiorly, with inspiration. Resonance on percussion, on both sides, at the summit, in front and behind, was clear and vesiculo-tympanitic; the sonorousness and tympanitic quality and elevation of pitch, being greater on the left side than on the right. On auscultation, sibilant and sonorous rales everywhere with expiration, and—to a less extent—with inspiration. At summit of chest, on right side, in front, short, *deferred* inspiratory sound; on same situation, on left side, *no sound* appreciable. Over the precordia, *clear* resonance on percussion; with the dry rales, on auscultation. No apex-impulse of heart, any where; sounds of heart heard most distinctly in the epigastrium. (Vide "Flint on Respiratory Organs, p. 445 and 447.") He was placed on the use of potassii iodidum, gr. v. 3 times daily, and increased to gr. x. 3 times, in a few days. A paroxysm of asthma occurred again in a few days. It will be unnecessary to trace this interesting case farther than this, up to a period when a false membrane was expelled from the bronchial tubes, except to notice the connection between the occurrence of asthmatic paroxysms, and atmospheric changes immediately preceding them; aud to glance at the variety and the efficacy of the remedies employed.

Dec. 14th, Dr. F. noticed the barometer to indicate an atmospheric change; on the preceding evening, prior to this, the weather had been pleasant; a storm of rain and snow came on during the night. The patient retired with no apparent difficulty of breathing, no cough, and no expectoration; about midnight, he was suddenly attacked with a paroxysm of asthma, moderate in severity, obliging him to maintain a semi-erect posture in bed; there were present lividity of the lips, capillary congestion of the hands, coldness of the skin, and frequent acts of coughing, attended by expectora tion of clear, striated mucus; after which he obtained relief. Passing by several paroxysms, for which reference must be made to the tabular statement appended to this case, on the 24th of Dec., another paroxysm occurred the preceding evening, under which he was suffering at the morning visit *Chloroform* was given by inhalation, freely, but not carried to complete anæsthesia. It gave the patient *marked* and *immediate* relief. The difficulty of breathing was much diminished; and the patient sank into a deep sleep. Previous to the employment of the chloroform, Dr. F. heard the sibilant rale *very distinctly* over scapular region on both sides; and after the administration of the chloroform, it was presumed to be *less intense.* Passing over another paroxysm in which the relief following the inhalation of this agent was as marked as it was in the preceding attack, on the 4th of Jan., the patient was found laboring under an asthmatic paroxysm which had come on during the night. At 10 A. M., the patient was still in the semi-recumbent posture in bed; the respirations were frequent and labored; during expiration, the sonorous rale could be heard at a distance from the chest; on placing the ear on the chest, the sonorous and the sibilant rales existed everywhere; and were musical in some situations. The patient entreated for chloroform; at 10½ A. M., it was given so as to produce nearly complete anæsthesia. The respirations became less frequent and less labored; the expiration was less prolonged; the sonorous rale could *not* be heard at a distance from the chest; the sonorous and sibilant rales were *very much diminished in intensity*, and the paroxysm disappeared about 1 P. M.

In order to bring before the reader, the number of paroxysms this patient experienced, their relation with preceding atmospheric changes, the results of the various remedial agents employed, a tabular statement is here appended.

*Remarks on Table.* It will be observed, that the paroxysms generally came on during the night or early in the morning; that they did not occur periodically; that they were variable as regards intensity, and were usually much more severe after a decided change in the atmosphere: almost all of these variations in the weather, were from a cold and dry atmosphere, to a warmer and moister atmosphere. It is also to be noticed, that no paroxysm occurred after or during certain decided atmospheric changes as great as those which, at other times, were followed or accompanied by asthmatic attacks. These paroxysms, even when most severe and when not influenced by remedial agents, did not continue longer than twenty hours.

The remedies employed were of two kinds, those intended to relieve *directly* the asthmatic attacks; and those intended to *prevent* their recurrence. Of the former class, the first three paroxysms were relieved in a measure, though not notably so, by the employment of repeated doses of a mixture of Syrup. Tolutani f. ʒ j, with *Morph. Sulph.* gr. $\frac{1}{4}$: this was given every twenty minutes, and appeared to produce *some degree of comfort.* This remedy was also used in the same dose, and with the same result, in three paroxysms numbered 8, 9, and 10 in the tabular statement. *Morphia*, uncombined with the syrup, was given in large doses, (gr. ss. every twenty minutes,) in one paroxysm; and with *more relief* than accompanied its use in the preceding attacks. Two paroxysms were allowed to terminate *without any immediate medication;* they continued about the same length of time as the attacks in which the mixture of tolu and morphia was given; there being no appreciable difference in this respect. The remaining medicines, with one exception, were administered by means of inhalation. The leaves of the *stramonium* were tried during the attacks of asthma; but the patient having made the attempt to inhale the smoke, could not be induced to repeat it; the effort occasioned him discomfort and increased the violence of coughing. After one of these attempts at inhalation, the patient was given a dose of *opium;* but it appeared to have no effect. The *nitrate of potassa* was employed once; coarse paper was dipped in a strong solution of nitre and dried; and then burned in such a manner that the smoke could be inhaled by the patient; the same reluctance to its use was entertained by him, although the small quantity of smoke he did inhale, gave him *slight relief.* In *six* of the paroxysms, most of them quite *severe*, the patient inhaled *chloroform;* it was never continued so long as to produce complete anæsthesia. It *always produced marked relief;* the difficulty of breathing and the frequency of the respiratory movements, were diminished; the cough ceased almost entirely; the patient could take the recumbent posture in bed; and sleep, undisturbed and profound, terminated such paroxysms; the physical signs also gave evidence of its beneficial effects; these results took place *immediately*, and the paroxysm was *much abridged.*

In regard to the remedies employed to prevent the recurrence of the asthmatic attacks, a few words may be said. The *iodide of potassium* was given for nearly nine weeks, and part of the time 30 grains daily were used; he took this medicine from the date of admission to the beginning of February; he suffered less from deficiency of breath than he had prior to entering the hospital; during the period he was exempt from asthmatic attacks; but this article *did not appear to diminish* the severity or the frequency of the paroxysm. After the iodide of potassium had been discontinued, the sulphate of quinia was used for a fortnight and appeared to produce no notable beneficial results. Fowler's solution was next administered and continued until the expulsion of the false membrane; no appreciable effect on the asthma, nor on the general condition of the patient, attended its use: it was given in such doses as to produce constitutional effects, as thirst, puffiness of eyelids, &c.

On the 18th of Feb., whilst laboring under a paroxysm of asthma, and during an act of coughing, "he expectorated, with much difficulty, a solid fibro-cartilaginoid substance about an inch in length, of a conical form; the base of the cone being nearly one quarter of an inch in diameter; the cone was hollow, with about one third of the circumference, in quite the entire length, wanting; the apex was somewhat blunted and not pervious; the walls of the cone were about two or three lines in thickness, and were creased longitudinally; it was opaque and extremely hard, and covered on its interior surface, with thick mucus. Immediately after the expectoration of this substance, the cough and labored breathing were relieved; and from this date to the 1st of March, a period of ten days, he had no cough, nor any marked difficulty of respiration, nor a paroxysm of asthma. About the middle of March, he commenced the use of the syrup of iodide of iron; and for two weeks, during which time the atmospheric changes were great and frequent, he did not suffer from any attack of asthma nor feel any inconvenience in breathing; he was daily out of doors, for several hours. This may be due to the expulsion of the false membrane, or to the change of season, or to the action of the syrup of iodide of iron."

*Remarks* (*a.*) Various opinions have been entertained by different observers respecting the cause of *emphysema* of the lung. Some have regarded it as depending on purely vital conditions, such as diminished elasticity of the pulmonary parenchyma; Laennec supposed that emphysema was produced by more or less occlusion of the finer bronchial tubes by mucus and the accumulation of air behind the seat of obstruction; others have thought that repeated and violent expiratory acts, or coughing, might give rise to emphysema. But, as yet, no satisfactory demonstration has been given of a diminished amount of elasticity or of tone in the air-vesicles; and expiratory efforts, though forcible, do not produce *mechanically* any other effect upon the air-cells, than to empty and contract them. As for the theory of the immortal discoverer of this morbid condition, it appears to be inconsistent with more recent and more extended observations; accumulation of mucus obstructing to a greater or less degree the bronchial tubes, has been found to produce, not dilation nor patescence of the air-vesicles, but collapse or contraction; besides this, the situations where mucus is much more often accumulated, are in the posterior and interior portions of the lung; whilst emphysema affects generally its anterior and thin edges. Dr. W. T. Gairdner, of Edinburgh, has lately advanced an entirely novel and ingenious theory, to account for the production of emphysema of the luug. In the outset, he lays down the following proposition as a law, "that collapse of the lung on becoming permanent, gives rise to a peculiar form of pulmonary atrophy;" and then he brings forward another statement, adducing the results of observation and well-known facts to support it. It is, "that collapse of the lung and pulmonary atrophy are the *invariable accompaniments* of emphysema of the lung: and the last mentioned condition depends essentially on one or both of the former two as its cause." He concludes, that "emphysema is a lesion of *mechanical* origin;" that "it is produced by the *inspiration-force* and *never during expiration;*" and that "it is due *solely and exclusively* to the action of that force upon the *previously sound air-vesicles* which has undergone in other portions, atrophy and collapse," that is, "partial diminution of volume." In the course of the argument, he makes use of the fact, in support of his proposition, that, the mechanical conditions of the lung, are such that it cannot expand, as a whole, or as one or more individual air-cells, beyond the normal extent produced by muscular agency; and hence if one or more portions have been increased in volume, other portions of the lung *must* have been previously *diminished in volume.* But, he believes that this diminution has been existing for some time prior to the development of emphysema. Nothing, however, militates against the idea that sometimes emphysema may be produced by dilatation of the air-vesicles occurring at or very soon after the beginning of collapse of other air-cells; may not the forces operating to bring about dilation, be the *cause* of the contraction or collapse of other portions of the pulmonary structure? The first of these three cases, seems to have been very recent in its appearance; and there is no evidence in the history of the case, to furnish us with any ground of suspicion of prior pulmonary difficulty. In the second case, however, the facts elicited favor the theory of Dr. G.; and so may the last case.*

Emphysema may exist uncomplicated, but generally it is associated with some bronchial affection, either varieties of bronchitis or spasm of the muscular fibres of the bronchi, producing asthma. In the first case, there was but little bronchial inflammation; in the second case, chronic bronchitis was present; and in the last case, plastic bronchitis and asthma.

The objects of treatment, then, are merely palliative, or preventive of an increase of the emphysema. In the first case, the *iodide of potassium* was employed in moderate doses, *with benefit.* In the second case, *no appreciable relief followed its use;* the *lac ammoniac*, however, was found always to produce *marked relief*, probably by reason of its influence on the bronchitis.

(*b.*) Asthma is now restricted to a "paroxysmal affection, the morbid condition of which consists in spasmodic contraction of the muscular fibrils of the finer bronchial tubes." The reader may find some interesting and applicable conclusions, in this connection, respecting asthma, in the last Dec. number of the Journal, p. 447, by Dr. Forget. The treatment of the case under consideration, has been already sufficiently commented on, in the remarks on the tabular statement appended to the last case.

(*c.*) Plastic or pseudo-membranous bronchitis is a rare form of disease. Dr. Watson mentions two cases, and Dr. Peacock has collected and analyzed forty-eight cases.† It is distinguished from other varieties of bronchitis, by the presence of a sub-crepitant rale limited to a certain portion of the chest, and by the characteristic expectoration. The disease consists in a fibrinous deposit or exudation on the mucus membrane of the smaller bronchial tubes; this exudation resembling the false membranes which occur in the larynx and trachea, in croup. Dr. P. draws the following, among other conclusions, from the analysis of the cases collected by him;

---

* Vide Brit. and For. Med. Chir. Review, April, 1853, for more extended discussion on the causes of emphysema of the lung.

† Vide American Jour. Med. Science, April, 1855, p. 499, *et seq.*

this disease is more frequent in males than in females; it occurs most frequently between the ages of 20 and 50; it is most commonly found in persons debilitated or not in robust health; it may be acute or chronic, partial or general, idiopathic or symptomatic.

The disease, in the present instance, did not call for remedies to relieve inflammatory symptoms, nor to assist in the expulsion of the membranes; but was directed more especially to the prevention of their farther formation, by means of tonics and of anodynes.

